# Effect of the Moisture Content of Recycled Aggregate on the Mechanical Performance and Durability of Concrete

**DOI:** 10.3390/ma15186299

**Published:** 2022-09-10

**Authors:** Daosheng Sun, Wei Huang, Kaiwei Liu, Rui Ma, Aiguo Wang, Yanmei Guan, Shansan Shen

**Affiliations:** Anhui Province Engineering Laboratory of Advanced Building Materials, Anhui Jianzhu University, Hefei 230022, China

**Keywords:** microhardness, pore structure, prewetted degree, recycled aggregate, strength

## Abstract

Wasted concrete was often used as a recycled aggregate instead natural stone in fresh concrete to reduce the environmental impact in a decade. However, because of the residual mortar interface, the performance of recycled aggregate was weaker. In this paper, the recycled aggregate was prewetted, and the effects of prewetted degree on the workability, strength, and durability of concrete were studied. The properties of the interfacial transition zone (ITZ), including microhardness, pore structure, and width, were also investigated. The results show that the workability intensity increased with the increase in prewetted degree from 0% to 100%, while the strength was first increased and then decreased with the optimal value of 43.3 MPa when the prewetted degree was 50–65%. The water absorption and chloride ion diffusion coefficient were also decreased by approximately 10% at minimum with the prewetted degree around 55% because of the declined fraction of pores larger than 50 μm and smaller porosity. The width of ITZ was first sharply decreased with a prewetted degree of 50–65%, then increased again with higher moisture, while microhardness of the ITZ showed the opposite trend and reached 82.7 MPa at maximum, at 50%. The appropriate moisture (50–65%) improved the pore structure and hydration products with an internal curing effect. When the moisture content was too high, the excess water was released from aggregate to the matrix, causing a higher water–cement ratio at ITZ; the porosity and the number of macrospores were increased to weaken the performance of concrete.

## 1. Introduction

With the rapid development of urbanization and industrialization, a huge amount of construction waste is generated every year [1,2]. In China, this number increased to 1.5–2.4 billion tons per year and reached 628 million tons in 2020, but the use of recycled construction waste was less than 5% [3,4]. Wasted concrete is a primary category of construction waste and often causes serious damage to soil, water resource, and human health when disposed of in landfills. Hence, using waste concrete as recycled aggregate to partially or totally replace natural aggregate in fresh concrete could effectively solve the problem of the shortage of natural resources [5,6], reduce the environmental impact, and save materials costs [7,8,9].

Compared with natural aggregates, the surface of recycled aggregates is usually attached to old mortar, so the physical properties, especially water absorption and crushing index, are significantly different [10,11,12,13,14,15,16,17,18,19]. The water absorption of recycled aggregate is usually approximately 3–8%, while the water absorption of natural aggregate is generally no more than 2% [13,15]. Moreover, with the attached mortar increase from 0% to 80%, the water absorption of recycled aggregate is continuously increased from 2% to approximately 10% [20,21,22,23,24,25,26,27,28,29]. Also, the crushing index of recycled aggregate is usually approximately 10–30% [10,11,12,13,14,15,16,17,18,19], which is generally higher than that of natural aggregate.

The old mortar attached to wasted concrete plays a key role in aggregate quality. Akbarnezhad et al. [25] found that when the amount of attached mortar increased from 14% to 58%, the compressive strength of recycled concrete decreased from 60 MPa to 50 MPa, because of the higher effective water–cement ratio and poor compactness caused by porous attached mortar. Moreover, the weak interfacial structure reduced the durability of recycled concrete. Otsuki et al. [29] found that when the content of attached mortar was 44.6%, the chloride ion penetration depth in recycled concrete was 1.2 times that of ordinary concrete. Using high-quality recycled aggregate with low water absorption and a small crush index, the water absorption and chloride ion permeability coefficient were significantly reduced. Afshar et al. [30] showed that the use of reshaped recycled aggregate reduced the capillary water absorption of recycled concrete and improved corrosion resistance. 

Since the wasted concrete with porous attached mortar increased the water absorption, which was harmful to the strength and compact microstructure of concrete, the absorbed water could also play the internal curing effect to improve the interface properties of concrete. Yang [31] proved that porous aggregates play the role of “internal curing” and refine the internal pore in concrete, which was less than 100 μm. Furthermore, the compressive strength and chloride ion penetration resistance of concrete were enhanced with the finer pores. Zhang [32] studied the effect of porous aggregate with a different prewetting degree on the performance of concrete and found that the internal curing effect was promoted by the prewetting degree. The hydration degree in the interfacial area, the polymerization degree of C-S-H gel, and the microhardness were all increased. Some scholars focused on the influence of recycled aggregates with different water contents on the performance of concrete [33,34,35,36,37,38,39]. The initial slump of recycled concrete was mainly determined by free water in concrete, while the slump loss value was affected by the water content of aggregates. Poon [33] set the recycled aggregate in three states—air-dried, oven-dried, and saturated dry-surface. It was found that when air-dried or oven-dried recycled aggregate was used, the slump loss of concrete reached the maximum. Additionally, the strength of air-dried recycled concrete was the largest at 3 d, 7 d, and 28 d. The compressive strength and splitting tensile strength of recycled concrete were improved with an optimal prewetting degree of recycled aggregate [30]. Cheng et al. [35] studied the performance of concrete with recycled aggregates containing moisture of 0%, 50%, and 100% and found that the mechanical performance was best when the moisture content was 50%. Mefteh [36] believed that saturated dry-surface recycled aggregates had the maximum negative impact on the strength of concrete, and the air-dried recycled aggregate optimized the strength of concrete. The water stored in the saturated surfaces of dry recycled aggregate was released into the slurry, increasing the effective water–cement ratio in the interfacial transition zone, resulting in an enlarged width and decreased mechanical properties and durability of recycled concrete [37]. Xu [38] studied the internal curing effect of recycled ceramic aggregate on high-performance concrete and found it led to better mechanical properties and reduced the autogenous shrinkage of concrete. Ge [39] found that saturated dry-surface recycled clay brick fine aggregates could limit the internal humidity and drying shrinkage of recycled concrete. The water-released effect of recycled brick aggregate provides enough moisture for cement hydration and maintains the internal humidity balance of concrete.

In this paper, recycled pure mortar aggregate was used to partially replace natural aggregate, and the moisture content of recycled aggregate was controlled by prewetting treatment. The effect of recycled aggregate with different moisture content on mechanical properties and durability was studied, and the interfacial structure of recycled concrete was investigated to reveal the mechanism of the internal curing effect of the prewetting recycled aggregate. The biggest highlight of this paper is exploring the effect of pure recycled mortar aggregate moisture content on concrete performance, which has a particular guiding significance for the application of low-quality recycled aggregate in concrete.

## 2. Materials and Methods

### 2.1. Raw Materials

**Figure 1:**
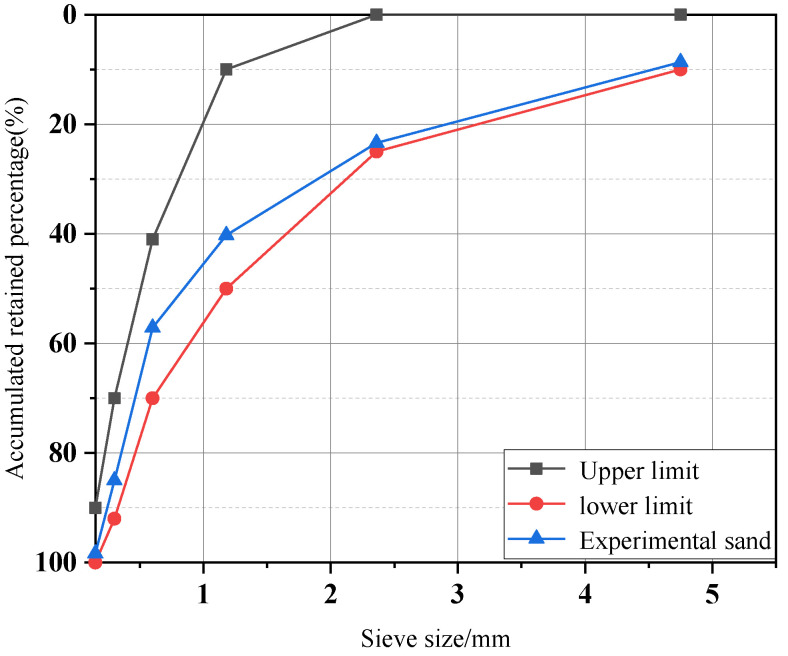
Screening curve of sand.

P·O 42.5 Portland cement and fly ash were used as binders. The chemical composition was tested by XRF and is shown in Table 1. The fine aggregate was natural river sand with a fineness modulus of 2.86; the sieved curve is shown in Figure 1. The polycarboxylic acid superplasticizer with a solid content of 50% was used to improve the workability.

**Figure 2:**
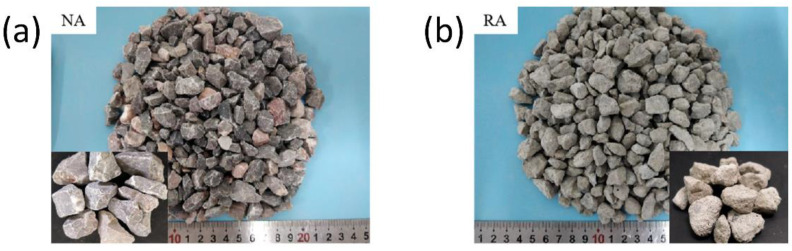
Morphology of the natural aggregate and recycled aggregate: (**a**) NA; (**b**) RA.

Natural stone and recycled concrete with sizes of 5 to 16.5 mm were both selected as natural coarse aggregate (NA) and recycled coarse aggregate (RA), respectively. The recycled aggregate was obtained from the crushed C30 concrete by a hummer crusher. The natural appearance of two coarse aggregates is presented in Figure 2. The natural stone had a smooth surface and angular shape, while the recycled aggregate had a more rounded shape and rougher surface. The basic physical properties of aggregates are listed in Table 2.

### 2.2. Methods and Procedure

The slump of recycled concrete was tested according to standard GB/T 50080 (test method for the performance of ordinary concrete mixtures) [40]. The compressive strength test was referred to as GB/T 50081 (standard for test methods of mechanical properties of concrete) [41]. Block samples (100 mm × 100 mm × 100 mm) were cured in a standard environment (20 ± 2 °C, RH > 95%) until the specified age for the test. During the test, the samples were loaded with a compact loading of 0.5 MPa/s to crush.

The water adsorption test was carried out according to ASTM C1585 [42]. The test piece was formed in a cylindrical mold with a diameter of 100 mm and a height of 50 mm. The concrete specimen was cured for 28 d, then dried at 50 ± 2 °C in a drying oven to constant weight. After cooling to room temperature, the sides of the specimen were covered with a layer of paraffin, and the top surface was sealed with plastic film to prevent water evaporation. The initial mass was recorded as *m*_0_. Then the concrete specimen was put into the water, keeping the distance between the bottom surface to the liquid level of 2 ± 1 mm. The mass *m_t_* of the specimen was recorded at the corresponding time point (1 min, 5 min, 10 min, 20 min, …, 192 h), and the water absorption per unit area of the concrete was calculated as follows:

(1) $$I=\frac{m_{t}}{A\times d}$$

where *I* is the water adsorption capacity per unit area in mm, *m_t_* is the mass of the test block at different times in g, *A* is the contact area between the test block and water, and *d* is the density of water, taken as 1000 g/mm^3^.

The electric flux test was followed by the standard method for the long-term performance and durability of ordinary concrete [43]. The cylindrical specimen was prepared with a diameter of 100 mm and a height of 50 mm. After curing to the specified age, the electric flux of the specimen was measured for 6 h.

The rapid chloride ion migration coefficient was determined according to the standard for test methods of long-term performance and durability of ordinary concrete [43]. A Φ100 mm × 50 mm cylindrical test piece was used to measure the chloride diffusion coefficient during unsteady and rapid migration.

**Figure 3:**
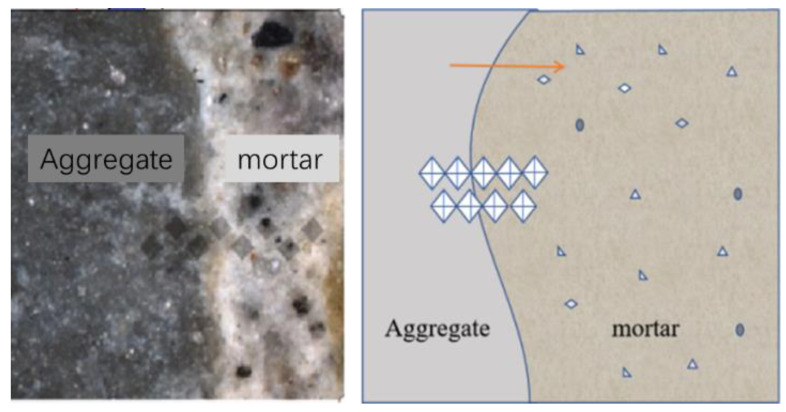
Schematic diagram of microhardness test. (arrow shows the direction of taking diamond shaped points).

A Dhv-1000 digital microhardness tester was used to test the microhardness changes in recycled concrete in the multiple interfacial transition zone (ITZ). The concrete slices cured for 28 d, with a thickness of 10 mm, were polished with 400, 800, 1000, and 2000 mesh sandpaper and vacuum dried at 60 °C to constant weight. The test load was 0.5 kN. The indenter was pressed into the surface of the test block and unloaded automatically after holding for 10 s. The prismatic indentation under a 400× microscope and the measurement of its diagonal length are shown in Figure 3; the measurement interval was 10 mm. The arrow shows the direction of taking points and the symbol of diamond is the schematic diagram of dots.

**Figure 4:**
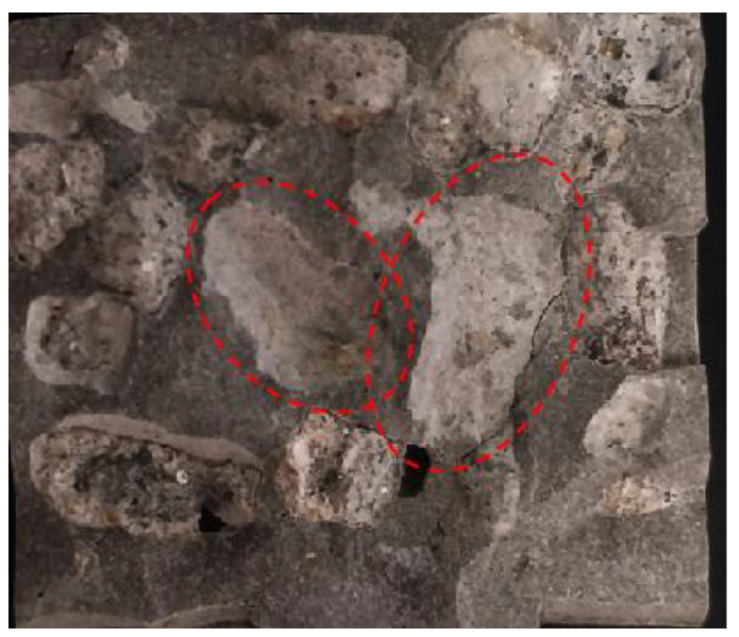
XRD interface sampling area. (red circle indicates the debonding area of the aggregate and mortar).

X-ray diffraction (XRD) was used to analyze the phase composition of the concrete interface; the cement mortar around the aggregate was scraped from tested concrete samples, as shown in Figure 4, and the red circle indicates the debonding area of the aggregate and mortar after peeling. The scraped powder was then ground to pass through a 200 mesh square sieve and vacuum-dried at 40 °C until constant weight.

After curing for 28 d, recycled concrete specimens were cut into cubes of 40 mm × 40 mm × 40 mm for porosity test using the nuclear magnetic resonance method. Small samples with aggregate and concrete matrix were prepared, cleaned in an ultrasonic cleaner for 5 min, and dried in a 45 °C vacuum-drying oven for 24 h. The micromorphology of the recycled concrete matrix and the interfacial area is observed by a GeminiSEM500 Schottky field emission scanning electron microscope produced by Carl Zeiss, Jena, Germany.

### 2.3. Mix Proportion

The proportion of recycled concrete was mainly obtained through a trial mix and further optimized based on previous research results. Recycled aggregate concrete was prepared with aggregates with different moisture contents. The moisture content of the saturated surface of dry (SSD) recycled aggregate was 100%, and the moisture content of recycled aggregate was controlled by spray pot prewetting and drying to be 50%, 55%, 60%, 65%, 75%, or 100%, respectively. Air-dry recycled aggregate with a moisture of 25% was the control group. Seven groups of recycled concrete with different moisture contents were prepared and denoted as AD, W50, W55, W60, W65, W75, and W100. The mix proportion details are listed in Table 3. The water–cement ratio was 0.45, the sand ratio was 45%, and the replacement ratio of recycled aggregate was 50% [44,45,46]. 

## 3. Results and Discussion

### 3.1. Aggregate Morphology Analysis

**Figure 5:**
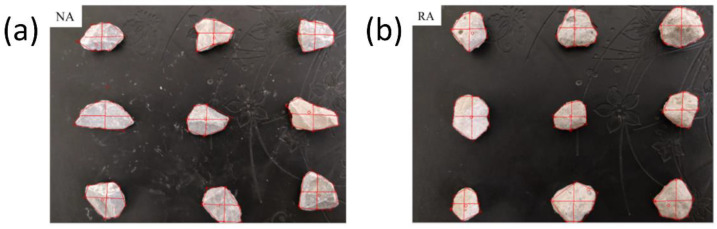
Image analysis of natural aggregate (**a**) and recycled aggregate (**b**).

**Figure 6:**
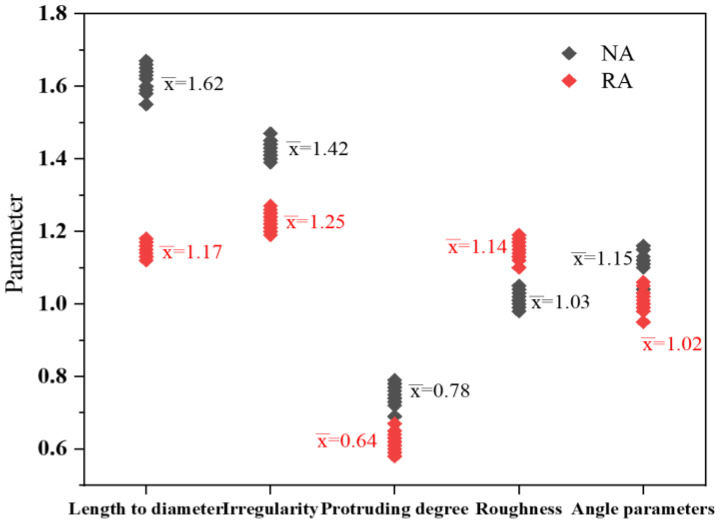
Statistics analysis on the parameter of natural aggregate and recycled aggregate.

According to the research method of aggregate characteristics [47], the morphological characteristics of natural aggregate and recycled aggregate were processed using an imaging method. Figure 5 is a schematic diagram of the aggregate image analysis method. The results show that the natural aggregate had smooth surfaces, larger aspect ratios, and obvious edges and angles, while the recycled aggregate had rougher surfaces and rounder overall shapes. Figure 6 is the parameter statistics of natural and recycled aggregates. Compared with natural aggregate, the length diameter ratio, irregularity, and corner parameters of the recycled aggregate were reduced by 27.8%, 12.0%, and 11.3%, respectively, but the roughness was improved by approximately 10.7%.

### 3.2. Aggregate Pore Structure

**Figure 7:**
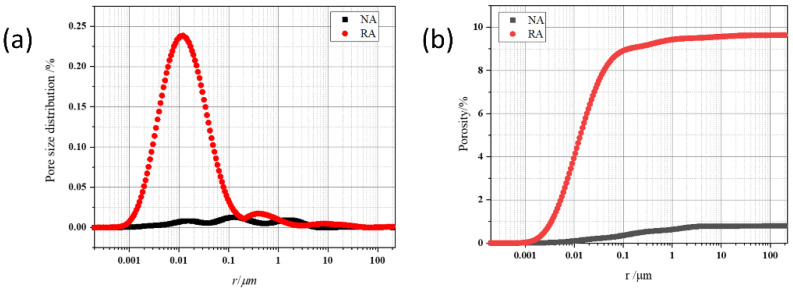
Pore size distribution (**a**) and cumulative porosity (**b**) of natural aggregate and recycled aggregate.

The internal pore size distribution and cumulative porosity of natural aggregate and recycled aggregate are shown in Figure 7, respectively. It was seen that the total porosity of NA was only 0.79%, implying that little open pores existed on the surface. In comparison, the porosity of RA reached 9.63%, which was approximately 12 times that of NA. In Figure 7a, a large fraction of pores with a diameter of 0.01 μm were detected in RA. The results also explained well why the water absorption of RA was much higher than NA.

### 3.3. Properties of Recycled Concrete

#### 3.3.1. Slump

**Figure 8:**
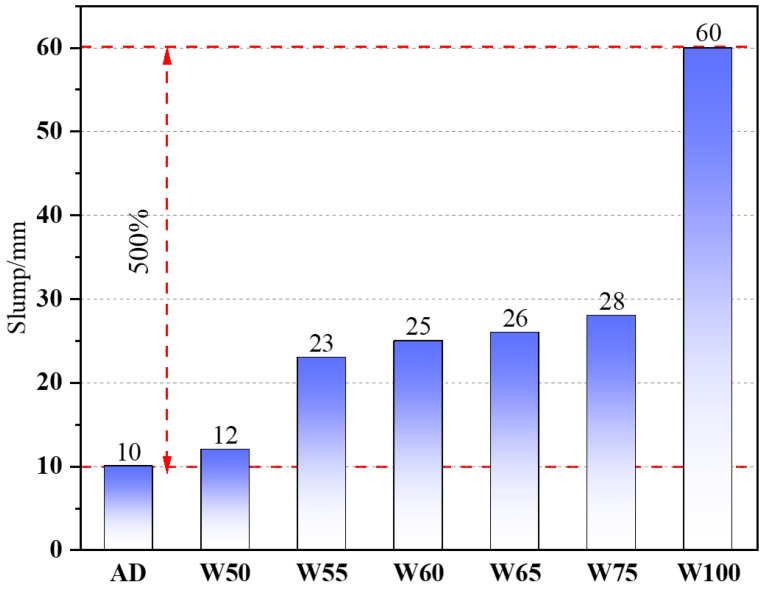
The slump of recycled concrete.

Figure 8 shows the influence of the prewetting degree of recycled aggregate on the workability of recycled concrete. When the moisture content increased from 0% (air-dry (AD)) to 100%, the slump of concrete continuously increased from 10 mm to 60 mm, which enlarged 500%.

The workability of concrete was sensitively affected by the free water in the fresh mixture. Since the water absorption of RA was higher than NA, more water was absorbed in wetting the RA particles during the mixing process, causing the decline of the real W/B ratio and an adverse effect on workability. So, with the prewetting degree increase in RA, the water absorption effect was weak; even the water in the aggregate was released into the mixture to improve the workability when the moisture content was exceeded. Relevant studies also showed that the slump of recycled concrete was the smallest when dry recycled aggregate was used, while it increased significantly when the recycled aggregate was saturated surface dry [48,49,50]. Since recycled aggregate usually had a porose surface, the different water content of aggregate changed the water absorption capacity, which sensitively affected the slump of recycled concrete.

#### 3.3.2. Compressive Strength

**Figure 9:**
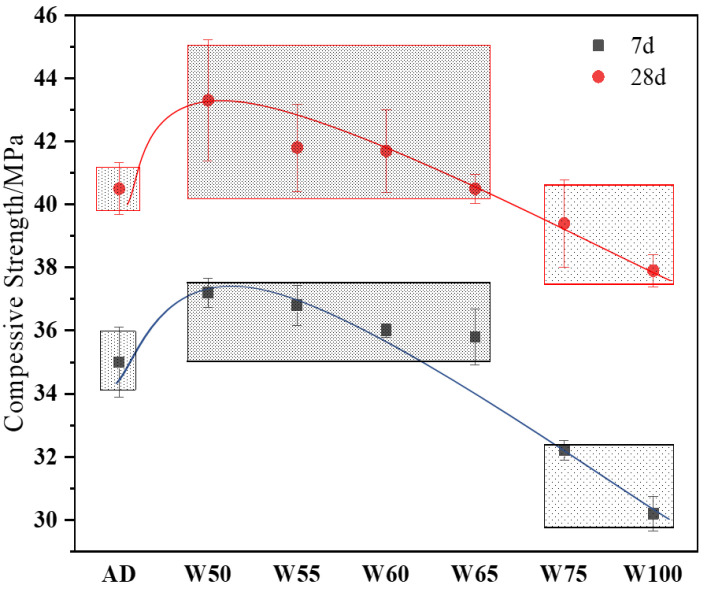
The compressive strength of recycled concrete at 7 d and 28 d.

The compressive strengths of recycled concrete at 7 d and 28 d with different prewetting aggregates are presented in Figure 9. From Figure 9, when the recycled aggregated was prewetted to 50% moisture, the compressive strength at both 7 d and 28 d was higher than the control group (AD). However, the value decreased when the moisture content of the aggregate further increased. At 7 d, the compressive strength of W50–W65 ranged from 37.2 to 35.5 MPa, which was higher than AD. With further prewetting from 75% to 100%, the strength became 32.2 and 30.2 MPa, decreased about 8% and 13.7% of AD. However, at 28 d, compared with AD, the compressive strength of W50–W65 was approximately 43.3–40.5 MPa, increased by about 6%. W75 and W100 decreased by 2.72% and 6.42% with the value of 39.4 MPa and 37.9 MPa, respectively.

From the results, the compressive strength of recycled concrete performed similarly at 7 d and 28 d, but the strength increments of prewetting aggregates were higher after 7 d. Since the recycled aggregate surface was porose and rough, it was supposed that if the moisture in aggregate was low, part of the mixed water was absorbed to form a water layer on the particle surface and reduced the real W/B so that the strength was increased. However, with the moisture of the aggregate raised, the water absorption effect was reduced, even the water was released from the aggregate into the matrix, causing a loose and weakened structure, and the strength was decreased. On the other hand, the absorbed water in the aggregate supports the internal curing to form more hydration products in the long term, so the strength increases more after an early age. Poon’s study found that the compressive strength of recycled concrete was highest when the recycled aggregate was in the dry state [33], while Brand believed that the strength was significantly improved by using partially prewetted recycled aggregate [34]. However, the W/B and the ITZ properties were the dominant factors for strength development. The moisture content which contributed to microstructure improvement in ITZ would benefit strength development.

#### 3.3.3. Capillary Water Adsorption

**Figure 10:**
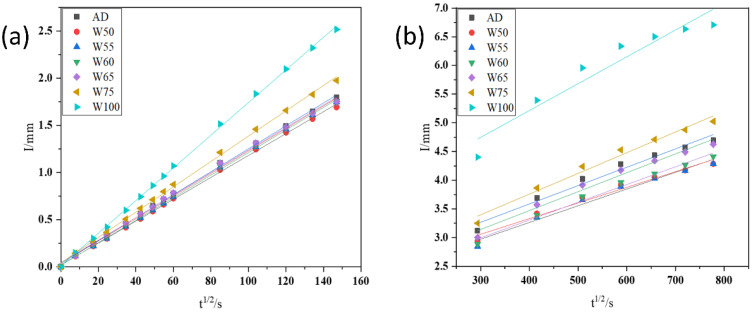
Water adsorption of recycled concrete at the initial time (6 h) (**a**) and secondary time (7 d) (**b**).

Figure 10 shows the initial water adsorption (a, 6 h) and the secondary water adsorption (b, 7 d) of recycled concrete. The water adsorption for all samples was linear and developed in both the initial time and secondary time. The capillary water absorption capacity was reflected by the slope of adsorption curves. In Figure 10, the capillary water absorption was first decreased and then increased with the moisture content of aggregate increased. Compared with AD, the capillary water absorption of W50–W65 continuously declined 1.5–8.9% at initial adsorption and 2.7–6.1% at secondary adsorption, respectively. However, when the moisture reached 75%, the water adsorption increased again. The capillary water absorption of W75 and W100 was 1.98 mm and 2.52 mm at 6 h, increased by 17.16% and 49.11%, while the value reached 5.02 mm and 6.71 mm at 7 d, with increases of 17.02% and 56.41%, respectively.

The main reason was that the effective water–cement ratio in the interfacial transition zone (ITZ) was adjusted by recycled aggregates of various moisture. When the water content was lower than 65%, mixed water was absorbed into aggregates, and the ITZ structure was compact and hard for water transport. However, if the moisture was exceeded, more water was released into ITZ, increasing the porosity and deteriorating the compactness so that the water adsorption was increased again.

**Figure 11:**
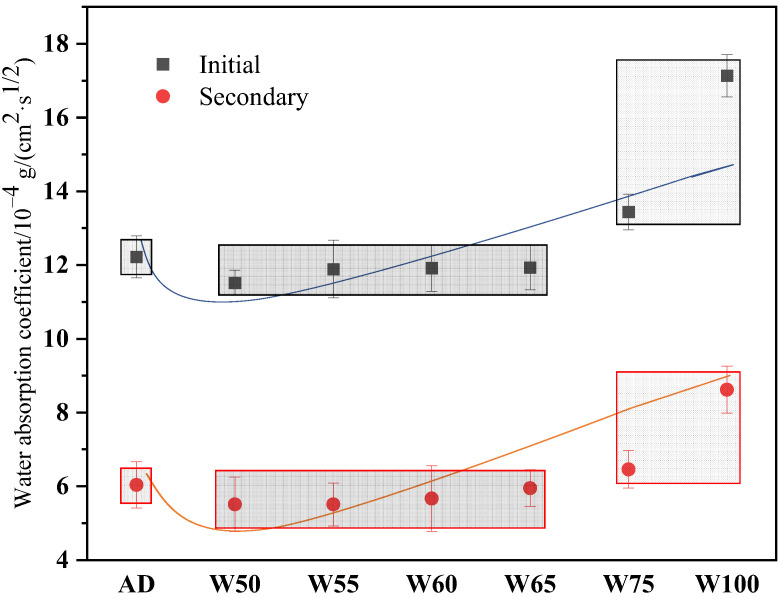
Water adsorption coefficient of recycled concrete.

The water adsorption coefficient of recycled concrete was also calculated and shown in Figure 11. The initial and secondary water adsorption coefficients of AD were 12.22 × 10^−4^ g/(cm^2^·s^1/2^) and 6.04 × 10^−4^ g/(cm^2^·s^1/2^), respectively. With the water content of prewetting aggregate increased, the initial and secondary water adsorption coefficients were reduced by 2.37–5.73% and 6.13–14.7%, respectively, when the moisture content was 50–65%, then increased by about 9.98% and 6.95% to 75%.

#### 3.3.4. Pore Structure

**Figure 12:**
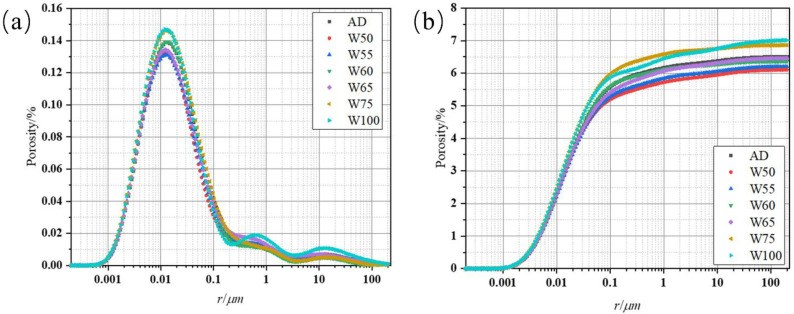
The pore size distribution (**a**) and cumulative porosity (**b**) of recycled concrete.

**Figure 13:**
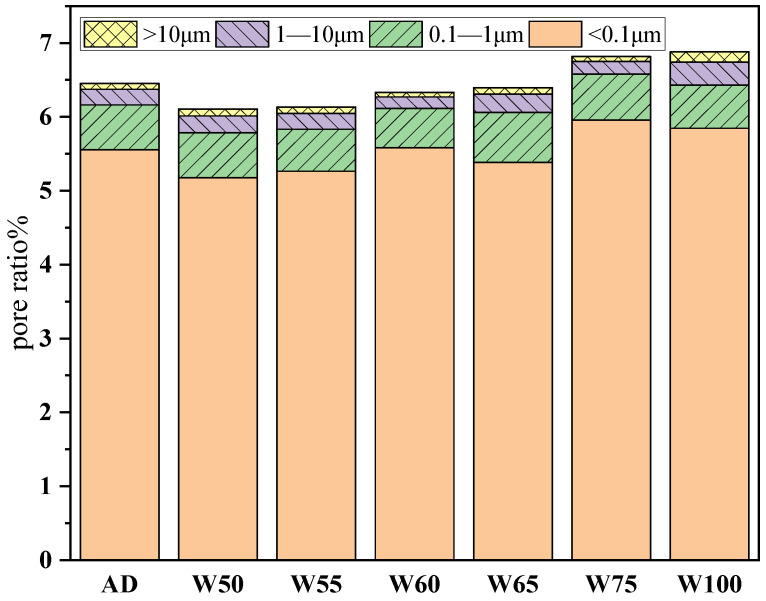
The proportion of pore diameter of recycled concrete.

To further investigate the change in the pore structure of recycled concrete with a different aggregate prewetting degree, the pore size distribution was measured by NMR, and the results are in Figure 12. The most probable pore diameter of recycled concrete was around 0.01 μm, and a small fraction of large pores existed with the size of about 1 μm and 10 μm. When the moisture content of the recycled aggregate ranged from 50 to 65%, the harmful pore diameter (greater than 50 nm) decreased; this value increased again in W100. Additionally, in Figure 12b, the cumulative porosity of recycled concrete with prewetting aggregate was lower than the dried one at the beginning, but the porosity increased from 6.10% to 7.01% with the water content rise from 50% to 100%, even higher than AD when moisture exceeded 75%. Figure 13 shows the proportions of the pore sizes of the recycled concrete. The proportion of pores larger than 1 μm on the saturated surface of dry recycled aggregate was significantly increased, accounting for 6.95% of the total porosity, and the number of macrospores increased significantly compared with AD. The results also proved that the aggregate with different prewetting degrees adjusted the pore structure of recycled concrete, and when the water content of aggregate was over 75%, absorbed water released into the matrix and generated more pores with large sizes. The results also corresponded with the strength development and water adsorption.

### 3.4. Durability of Recycled Concrete

#### 3.4.1. Electric Flux

**Figure 14:**
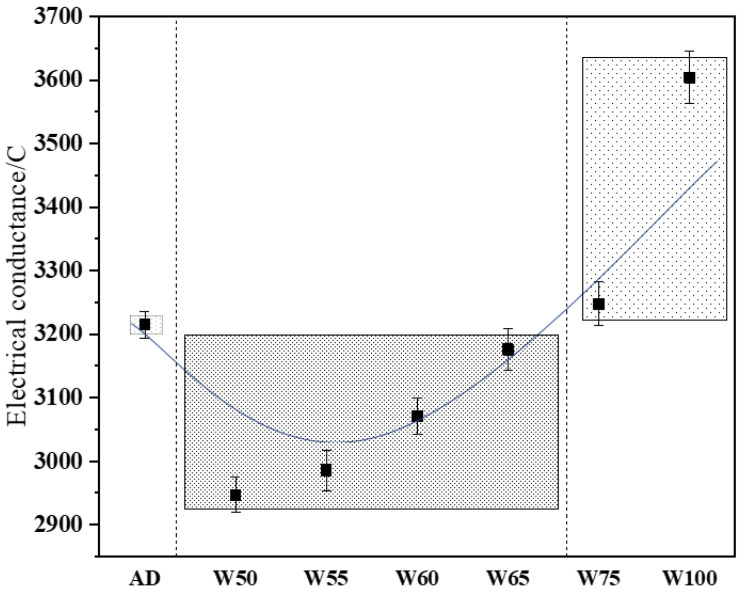
The electric flux of recycled concrete.

Figure 14 shows the electric flux of recycled concrete for 28 d. The results showed that when the moisture content of recycled aggregate was 50%, the electric flux was sharply decreased to the control group and reached the smallest value of 2947 C. With the increase in prewetting degree, the electrical conductance continuously increased, and when the moisture was 75%, it exceeded the control one (AD). The 28 d electrical conductance of W100 reached 3604 C, increased by about 10.8%, compared with AD. The use of saturated surface dry recycled aggregate increased the effective water–cement ratio in ITZ, and the loose and porous structure of the interfacial transition zone provided a convenient channel for chloride ion transport.

#### 3.4.2. Chloride Ion Permeability Coefficient

**Figure 15:**
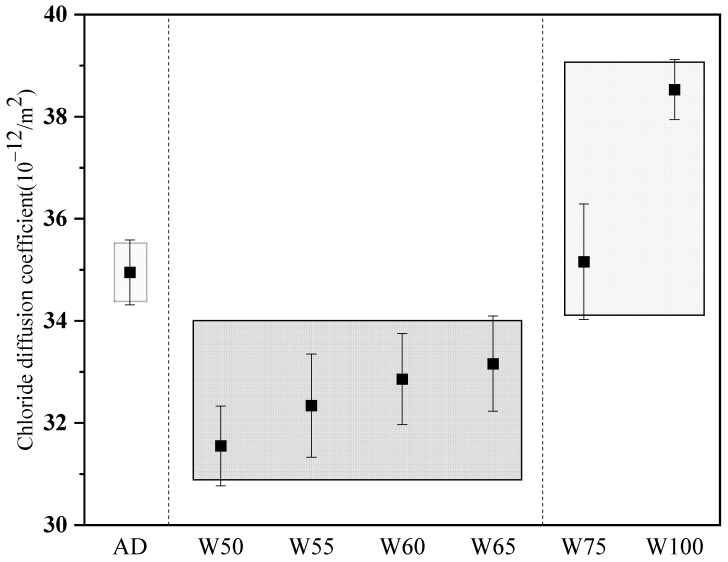
28 d chloride diffusion coefficient of recycled concrete.

Chloride penetration is the main factor causing steel corrosion in concrete. The effect of aggregate moisture on chloride penetration was evaluated by chloride ion diffusion coefficient, and Figure 15 shows the results of recycled concrete curried after 28 d. It can be seen that the change in chloride ion diffusion coefficient with different aggregate moisture was similar to electric flux. The value first decreased then increased with the increasing moisture content, and the sample of W50 performed the lowest diffusion coefficient of 10.55 × 10^−^^12^ m^2^/s, which was lower by about 10% than that of AD. Once the moisture reached 100%, the diffusion coefficient increased to 38.56 × 10^−12^ m^2^/s, which was 10.3% higher than in AD. Zhao et al. [39] also proved that the chloride ion diffusion coefficient of recycled concrete with saturated surface dry aggregate was larger than air-dried one because of the weak microstructure in ITZ.

### 3.5. The Performance of ITZ

#### 3.5.1. Microhardness

**Figure 16:**
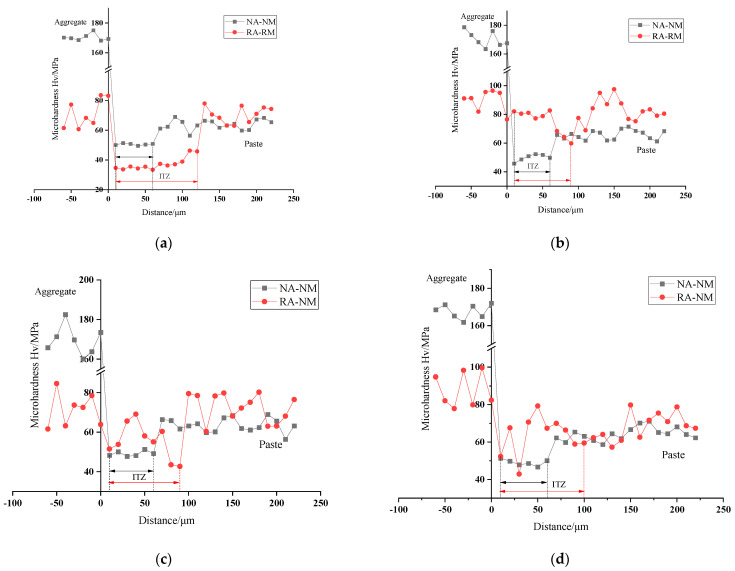

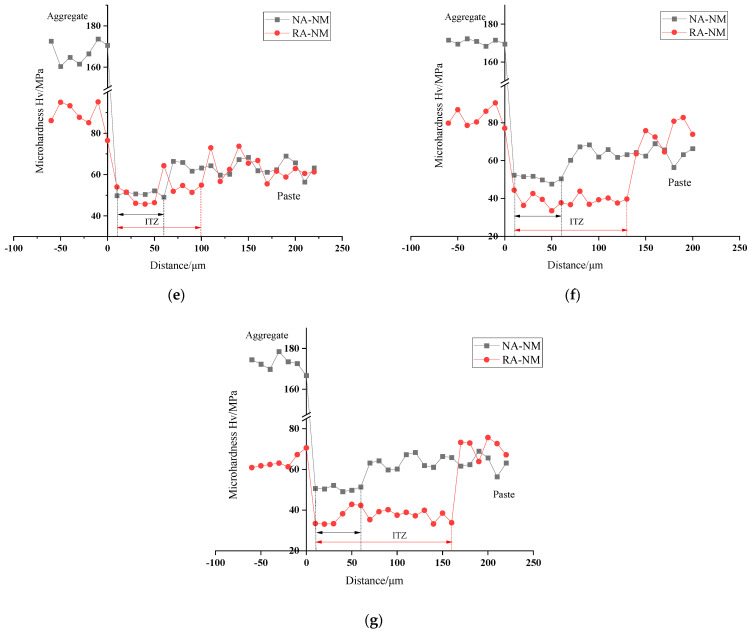
Microhardness of the concrete interfacial transition zone of recycled concrete: (**a**) AD; (**b**) W50; (**c**) W55; (**d**) W60; (**e**) W65; (**f**) W75; (**g**) W100.

Microhardness can be used to quantitatively analyze the micromechanical properties and width of ITZ of recycled concrete. The microhardness around ITZ of recycled concrete with natural aggregate (NA-NM) and recycled aggregate (RA-NM) was comparatively studied and shown in Figure 16. Since the microhardness of ITZ was typically smaller than the aggregate and the matrix, the change could help us to determine the boundary of ITZ and was marked in Figure 16; the average microhardness of ITZ is listed in Table 4.

In Figure 16a, once the aggregate was dried, the width of ITZ with natural aggregate (NA-NM) was approximately 60 μm, which was smaller than that of recycled aggregate (RA-NM) at 120 μm. Still, the microhardness of ITZ in RA-NM was only 33.4–45.8 MPa, and lower than that of NA-NM. However, after proper prewetting treatment, the width of ITZ suddenly decreased, then slightly increased with the degree of prewetting. The width of ITZ in W50–W65 was 90–100 μm, and when using the saturated surface dry aggregates, this value increased to 160 μm. On the other hand, the microhardness showed the opposite change, which sharply increased in W50 and declined with the rise of aggregate moisture. The microhardness ranged at 42.9–82.7 MPa in W50 and continuously decreased to 33.3–42.8 MPa in W100. It was commonly accepted that ITZ was the weak area in concrete; the results proved that, with a moisture content of 50–65%, the microhardness of ITZ was stronger than that of natural aggregate, which improved the mechanical performance and durability of recycled concrete.

#### 3.5.2. XRD

**Figure 17:**
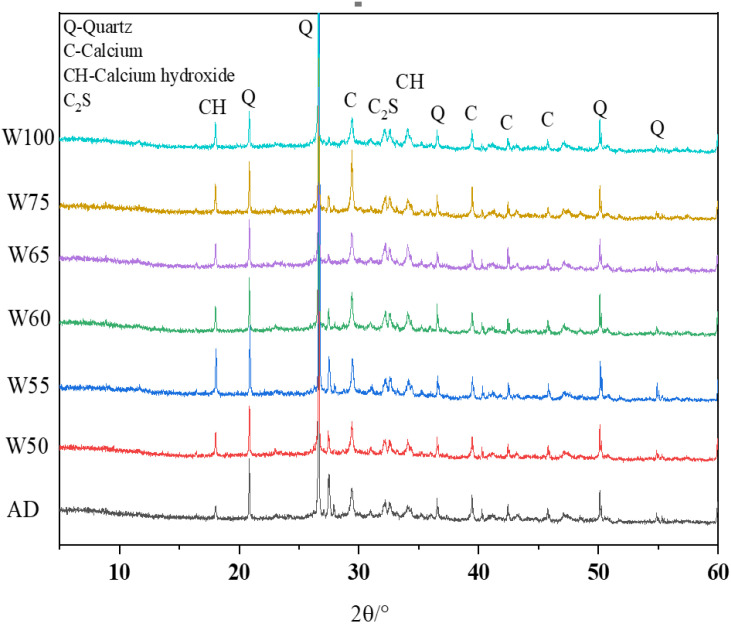
XRD spectra of the interfacial transition zone of recycled concrete.

The XRD patterns from ITZ of recycled concrete with different prewetting aggregates are presented in Figure 17. The diffraction peaks at 18.1° and 34.1° belong to the main products of Ca(OH)_2_ in ITZ. The diffraction peaks of CaCO_3_ appeared at 29.4°, 39.4°, and 43.15°, which might be caused by carbonization. The diffraction peaks are marked in Figure 17. The peak positions in each sample were not changed, indicating that the hydration reaction with different moisture was not changed with the same kind of hydration products.

#### 3.5.3. SEM

**Figure 18:**
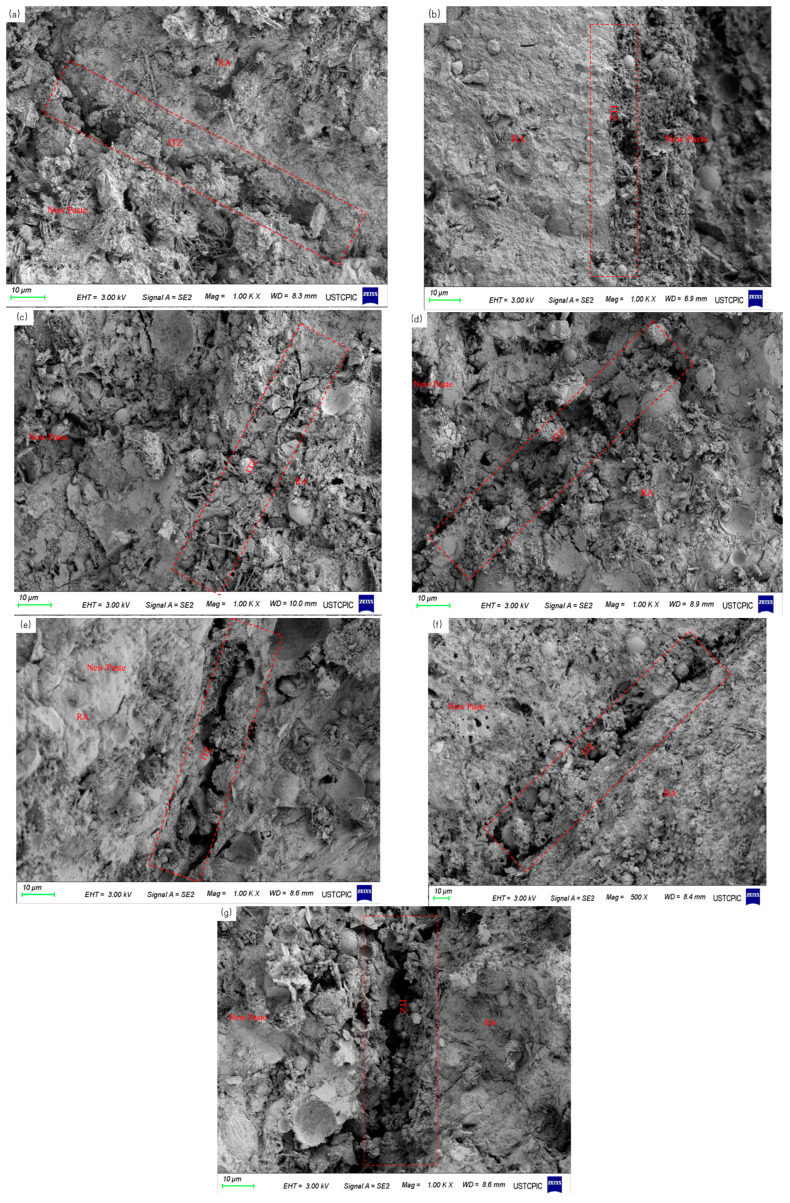
SEM images of the interfacial transition zone of the recycled concrete: (**a**) AD; (**b**) W50; (**c**) W55; (**d**) W60; (**e**) W65; (**f**) W75; (**g**) W100.

The morphology of ITZ in recycled concrete was observed by scanning electron microscopy (SEM) and presented in Figure 18. It can be found that the combination of aggregate and new mortar was poor and obvious cracks existed in ITZ. However, compared with AD, the ITZ of W50 to W60 was more compacted with fewer cracks, and the crack width of ITZ of W65 was similar. With the moisture content further increasing, the microstructure of ITZ became weak again, and larger cracks were found.

The moisture migration of recycled aggregates with different prewetting degrees followed the different laws at the early mixing stage [51]. When the content of the aggregate is lower than 50%, the internal humidity was low. A large amount of water from the slurry is rapidly absorbed by the aggregate, thereby reducing the actual mixing water and slump of the concrete. In addition, the adsorbed water initially accumulated on the aggregate surface and formed a water layer, causing the increase in the effective water–cement ratio in ITZ. With the prewetting degree increase, the width of the ITZ increased, the microhardness decreased obviously, and the compressive strength of the recycled concrete also decreased. The high porosity of the interface led to an increase in the capillary water adsorption rate and a decrease in the chloride ion corrosion resistance of the recycled concrete. Once the recycled aggregate was properly prewetted(approximately 50–65%). The surface humidity of recycled aggregate is high, which slowed down the rate of water absorption from the slurry to a certain extent and reduced the total amount of water absorption. So, the slump of the recycled concrete showed a slightly increase. The surface of recycled aggregate maintained a certain wet state, the water layer did not form easily on its surface, the effective water–cement ratio at the interface decreased slightly, and the width of the interfacial transition zone was significantly reduced to approximately 90–100 μm. The microhardness was in the range of 42.7–82.7 MPa. Compared with AD, the interfacial structure was effectively improved, which benefited compressive strength. The porosity of the recycled concrete was also reduced (0.77–6.30%) and smaller than that of AD. The appropriately prewetted recycled aggregate can play a certain role in internal curing, adding enough moisture to the concrete matrix for a full hydration reaction and improving the internal structure of the concrete.

However, when using saturated dry recycled aggregate, the water stored in the aggregate was released into the slurry under the action of centrifugal force, and the effective water–cement ratio of the interfacial transition zone and matrix increased. The high water–cement ratio at the interface led to an increase in the width of the ITZ and a decrease in microhardness and compressive strength. At the same time, the internal porosity of the recycled aggregate concrete with high moisture content was larger, and it was much higher than in the groups with slow and appropriate levels of prewetting, reaching 7.01%. The water absorption rate of concrete capillary water increased, and external ions were more likely to invade the interior of concrete and the chloride ion erosion resistance of recycled concrete.

## 4. Conclusions

The physical performance of recycled aggregate was less than that of natural stone. To improve the properties of recycled concrete, the aggregate was prewetted to various degrees, and the results proved that the strength, impermeability, and ITZ properties were all improved at appropriate prewetted degrees. Below are some conclusions:

(1) Recycled aggregate made from crushed concrete had a rough surface, round particle shape, and higher porosity than natural aggregate. The water absorption and crushing index were 25 times and 2.5 times higher than those of natural aggregate;
(2) With the prewetted degree continuously increasing from 0% to 100%, the slump of concrete was enlarged by 500%, while the strength was higher than the plain group at 50–65% and reached the maximum of 40.5 MPa. The appropriate moisture content of recycled aggregate would balance the water absorption in concrete at an early age, and more water would be released into the matrix to increase the real W/B ratio if the prewetted degree was too high;
(3) The permeability of recycled concrete was also first decreased and then highly increased with the prewetted degree of aggregate increasing from 0% to 100%, and was optimal when aggregate was prewetted to 50–55%. The internal curing effect of wet aggregate declined the porosity by 0.77–6.30% and reduced the fraction of pores larger than 50 nm. When the moisture increased further, the porosity increased significantly;
(4) The microhardness demonstrated that the width of the ITZ of recycled aggregate was larger than natural aggregate. However, with the prewetted degree of 50–60%, the width decreased to 90–100 μm, and the microhardness in ITZ ranged at 42.7–82.7, which was even higher than that of natural aggregate. The morphology also proved that with the prewetted degree of 50–60%, the ITZ was more compact with fewer cracks than another status;
(5) Prewetted degree influenced the driving force of water absorption at an early age and the internal curing efficiency. At an appropriate prewetted degree, the water absorption was mitigated, thinner water film formed at the aggregate surface, which caused a compact microstructure formation, and the water in the aggregate migrated gently to promote the hydration by internal curing effect. Exceeded water might rapidly release into the matrix, increasing the porosity and causing a loose ITZ, which was weak for strength and impermeability.

## Figures and Tables

**Table 1:** Chemical composition of cement and fly ash (mass fraction/%).

Material	CaO	SiO_2_	Al_2_O_3_	Fe_2_O_3_	MgO	Na_2_O	SO_3_
Cement	69.00	17.90	3.92	3.42	0.79	0.12	3.43
Fly ash	4.28	51.17	32.12	5.25	0.95	0.50	0.91

**Table 2:** Physical properties of natural aggregate and recycled aggregate.

	Bulk Density (kg/m^3^)	Apparent Density (kg/m^3^)	Void Absorption%	Water Absorption%	Crush Index %
NA	1580	2740	42.3	0.38	6.13
RA	1210	2570	52.9	9.75	15.27

**Table 3:** Mix proportion of recycled concrete (kg/m^3^).

	Water	Cement	Fly Ash	Natural Aggregate	Fine Aggregate	Recycled Aggregate	Prewetting Water	SP
AD	198	352	88	532	820	468	0(AD)	0.44
W50	198	352	88	532	820	468	3.60	0
W55	198	352	88	532	820	468	5.09	0
W60	198	352	88	532	820	468	6.58	0
W65	198	352	88	532	820	468	8.07	0
W75	198	352	88	532	820	468	11.04	0
W100	198	352	88	532	820	468	29.76(SSD)	0

AD—air-dried, SSD—saturated surface dry.

**Table 4:** The variation range of 28 d microhardness in the interfacial transition zone of recycled concrete.

	AD	W50	W55	W60	W65	W75	W100
NA-NM	49.6–51.4	45.7–52.3	47.8–51.3	46.7–52.3	49.8–52.2	47.6–52.3	49.1–52.2
RA-NM	33.4–45.8	59.8–82.7	42.7–69.1	42.9–72.3	45.7–64.3	33.5–44.4	33.3–42.8

## Data Availability

Not applicable.

## Abbreviations

Abbreviations		Symbols	
C-S-H	Calcium silicate hydrate	*I*	Water adsorption capacity
XRF	X-ray fluorescence	*m_t_*	Mass at time t
NA	Natural aggregate	A	Contact area
RA	Recycled aggregate	d	Density of water
ITZ	Interfacial transition zone		
XRD	X-ray diffraction		
SSD	Saturated surface dry		
AD	Air-dried		
W/B	Water to binder ratio		
RH	Relative humidity		
